# Biochemical Differences in Cerebrospinal Fluid between Secondary Progressive and Relapsing–Remitting Multiple Sclerosis

**DOI:** 10.3390/cells8020084

**Published:** 2019-01-24

**Authors:** Stephanie Herman, Torbjörn Åkerfeldt, Ola Spjuth, Joachim Burman, Kim Kultima

**Affiliations:** 1 Department of Medical Sciences, Clinical Chemistry, Uppsala University, 751 85 Uppsala, Sweden; Stephanie.herman@medsci.uu.se (S.H.); Torbjorn.akerfeldt@akademiska.se (T.Å.); 2 Department of Pharmaceutical Biosciences and Science for Life Laboratory, Uppsala University, 751 24 Uppsala, Sweden; Ola.spjuth@farmbio.uu.se; 3 Department of Neuroscience, Uppsala University, 751 85 Uppsala, Sweden; Joachim.burman@neuro.uu.se

**Keywords:** multiple sclerosis, cerebrospinal fluid, metabolomics, mass spectrometry, tryptophan, pyrimidine

## Abstract

To better understand the pathophysiological differences between secondary progressive multiple sclerosis (SPMS) and relapsing-remitting multiple sclerosis (RRMS), and to identify potential biomarkers of disease progression, we applied high-resolution mass spectrometry (HRMS) to investigate the metabolome of cerebrospinal fluid (CSF). The biochemical differences were determined using partial least squares discriminant analysis (PLS-DA) and connected to biochemical pathways as well as associated to clinical and radiological measures. Tryptophan metabolism was significantly altered, with perturbed levels of kynurenate, 5-hydroxytryptophan, 5-hydroxyindoleacetate, and *N*-acetylserotonin in SPMS patients compared with RRMS and controls. SPMS patients had altered kynurenine compared with RRMS patients, and altered indole-3-acetate compared with controls. Regarding the pyrimidine metabolism, SPMS patients had altered levels of uridine and deoxyuridine compared with RRMS and controls, and altered thymine and glutamine compared with RRMS patients. Metabolites from the pyrimidine metabolism were significantly associated with disability, disease activity and brain atrophy, making them of particular interest for understanding the disease mechanisms and as markers of disease progression. Overall, these findings are of importance for the characterization of the molecular pathogenesis of SPMS and support the hypothesis that the CSF metabolome may be used to explore changes that occur in the transition between the RRMS and SPMS pathologies.

## 1. Introduction

Multiple sclerosis (MS) is a common neurological disease. At onset, most patients are diagnosed with relapsing-remitting MS (RRMS). Over time, many will progressively accumulate disability, presumably due to a neurodegenerative process. This condition is called secondary progressive MS (SPMS) [1]. The gradual transition between RRMS and SPMS makes it difficult to recognize at onset, so SPMS is currently diagnosed retrospectively.

The pathophysiology of RRMS and SPMS is different and these conditions will probably require different treatments. Many disease-modifying treatments are currently available for RRMS patients [2], but only one (Ocrelizumab) has shown efficiency in treating progressive MS [3,4]. The need right now is to accurately distinguish between these two phenotypes and identify markers of disease progression. This would enable more stringent characterization of the pathophysiology of MS phenotypes, as well as improve selection of patients that are suited for certain clinical trials or interventions.

Metabolomics is a comprehensive profiling of the dynamic molecular networks composed of low-weight molecules or metabolites. These metabolites essentially correspond to the intermediate and end products of ongoing pathophysiological processes. Different metabolomic technologies have been used to study pathophysiology in MS [5], mainly focusing on the blood [6,7,8,9,10,11]. Pathological changes occurring in the central nervous system (CNS) are mirrored in the cerebrospinal fluid (CSF), which makes the CSF an attractive source to study [5]. Nuclear magnetic resonance spectroscopy-based methods have previously been used to study the CSF metabolome, comparing MS with controls and other neurological diseases [12,13,14,15,16,17]. Using high-resolution mass spectrometry (HRMS), we recently demonstrated that CSF metabolites integrated with protein and magnetic resonance imaging (MRI) information can improve early detection of SPMS [18].

The aim of this study was to get a better understanding of the pathophysiological differences between the SPMS and RRMS phenotypes. We profiled biochemical alterations in the CSF metabolome of SPMS patients compared with RRMS and controls using HRMS and multivariate statistics. The metabolomic differences were further connected to biochemical pathways and clinical data that revealed tryptophan and pyrimidine metabolisms to be of particular interest for understanding the disease mechanism and their metabolites as potential markers of disease progression.

## 2. Materials and Methods

### 2.1. Ethics Approval

The study was approved by the Regional Ethical Board of Uppsala (DNr 2008/182). All subjects provided written informed consent.

### 2.2. Subjects

Subjects (*n* = 56) were recruited from the Uppsala University hospital, where 30 participants were diagnosed with RRMS, 16 with SPMS and 10 were controls with other, non-inflammatory, neurological diseases (e.g., idiopathic intracranial hypertension or thunderclap headache). All MS patients met the revised McDonald’s criteria for MS diagnosis [19]. Seventeen of the RRMS and two of the SPMS patients were actively inflammatory, with the presence of gadolinium enhancing lesions on MRI.

All participants underwent a clinical examination with scoring in the Expanded Disability Status Scale (EDSS) and a lumbar puncture at inclusion. To record disease activity, MRI was performed within a week of the lumbar puncture at 1.5 T using the same imager and imaging protocol in all examinations. Gadopentetate dimeglumine (Magnevist^®^, Bayer AB, Solna, Sweden, 0.4 mL/kg body weight, i.e., double dose) was used as a contrast agent and MR images were analyzed visually. A detailed description of the MRI investigations has been published previously [18,20].

### 2.3. Sample Collection

The lumbar puncture was performed through the L3/L4 or L4/L5 interspace and CSF was collected in accordance with the guidelines formed by the BioMS-eu network [21].

### 2.4. Metabolite Extraction

The protocols for metabolite extraction and mass spectrometry analysis have been previously published by us [18]. Briefly, metabolites were extracted using ice-cold methanol (MeOH), supplemented with a cocktail of internal standards that was added to 100 µL of CSF (thawed on ice). After extraction, the samples were dried and reconstituted in 100 µL of 5% MeOH, 0.1% formic acid, and 94.9% deionized MilliQ water upon analysis. Ten microliters of each sample were pooled to create a quality control (QC) sample that can be injected repeatedly throughout the analysis.

### 2.5. Mass Spectrometry Analysis

Ten microliters of each sample were injected in a constrained randomized order into a Thermo Ultimate 3000 HPLC equipped with a Thermo Accucore aQ RP C18 column (100 × 2.1 mm, 2.6 µm particle size) and coupled to a Thermo Q-Exactive Orbitrap (all purchased from Thermo Fisher Scientific, Hägersten, Sweden). The mass spectrometer was operated in positive and negative ion mode and MS resolutions were set to 70,000 at *m*/*z* 200, AGC target 1,000,000, and maximum ion injection time 250 ms. A QC and a blank injection were done every eighth sample. Finally, a 2-fold serial dilution series ranging from 0.5 to 32.0 µL QC was injected. For improving metabolite identification, eight tandem mass spectrometry analyses in both ion modes were performed separately on pooled samples stratified on the diagnostic groups.

### 2.6. Quantification

The acquired raw data were converted to an open-source format (.mzML). Peak picking was performed using *msconvert* from ProteoWizard [22] and preprocessing using the following pipeline within the KNIME platform [23]. The peak-picked data were quantified by FeatureFinderMetabo [24] and the resulting features were linked across the samples using FeatureLinkerUnlabelledQT [25]. The time tolerance was set to 10 s and a 5 ppm mass deviation was allowed. The non-default parameters can be found in Table S1.

The quantified data were loaded into the statistical software environment R v3.4.0 [26]. Contaminants were removed first by using the blank injections, according to our previously introduced pipeline [27], and secondly by only keeping the metabolites that achieved an absolute Pearson correlation of 0.7 or higher between the relative abundances and injection volumes in the dilution series. To stabilize variance, the intensity values were replaced by the log^2^ value and potential sample outliers were detected and removed by calculating the total ion count (TIC) of each sample. Samples with a TIC less than 60% of the average TIC were seen as outliers and removed from the study. No sample outliers were removed.

All metabolic features with a 75% coverage were matched against an *in-house* library of characterized metabolites using a 15 ppm mass tolerance and a 20 s time window. Only metabolic features that matched a metabolite in the library were kept. To correct for potential intensity decay throughout the analysis, LOESS curves were fitted for each metabolite using the R function “loessFit” from the R-package *limma* and a span of 0.3, which were used for normalization [28]. To assess the robustness of the metabolites, the coefficient of variation (CV) was calculated on inverse log^2^ values for each identified metabolite in the QC samples. Thereafter, the in-between-replicate correlation was calculated (minimum replicate correlation achieved was 0.99) and the replicates averaged. Spearman’s rank correlation coefficients were calculated between the albumin ratio and the metabolites to eliminate compounds that may originate from blood (leaking through the blood‒brain barrier). Metabolites that acquired a statistically significant (*p*-value < 0.05) absolute correlation higher than 0.5 were removed. Finally, the remaining missing values were replaced by the average metabolite value.

### 2.7. Metabolite Identification

Metabolites (identified metabolic features) of interest were semi-automatically curated on MS/MS fragmentation level when available. MS/MS peaks were extracted and matched to the corresponding fragmentation pattern from the *in-house* library, where ≥50% coverage or at least five MS/MS peaks in common, as well as a dot product score above 0.5, was seen as a match. Identities confirmed by *m*/*z* and elution time of the pure standards and by MS/MS fragmentation pattern were depicted as verified on validation level 2. Identities confirmed only by *m*/*z* and elution time of the pure standards were depicted as verified on validation level 1. Identities of metabolites with an available MS/MS fragmentation that did not match the fragmentation pattern of the pure standard were rejected.

### 2.8. Statistical Analysis

Post hoc comparisons were performed for age, gender, EDSS, and disease duration for SPMS and RRMS patients. For variables not significantly different from a normal distribution, Welch’s *t*-test was applied, otherwise the non-parametric Mann‒Whitney test was used. The Shapiro‒Wilk normality test was used to assess normality of variables. For the categorical variable gender, a Chi-squared test was used. A *p*-value < 0.05 was considered statistically significant. To investigate treatment status as a potential confounder, a partial least squares discriminant analysis (PLS-DA) model was trained on all MS patients, discriminating between patients with ongoing treatments versus patients without treatments.

Correction for age was done using linear detrending based on RRMS patients and controls. Metabolic features with a significant age dependence (*p*-value < 0.05) were corrected by fitting a linear regression model (R function “lm”) for the metabolite levels in RRMS patients and controls, with age as the explanatory variable. The age coefficient was extracted from the model and used to correct the metabolic levels in all individuals [29].

To target inter-group differences, supervised multivariate analysis using PLS-DA was performed on identified metabolites. The data were scaled (zero mean, unit variance) and three PLS-DA models were trained, comparing two groups at a time using the R package *ropls* [30]. The most significant variables were obtained using the “Variable Importance in the Projection” (VIP). To assess model performance, the quality metrics *R*^2^ and *Q*^2^ were extracted and the area under the receiver operating characteristic (AUROC) was computed using the R package *pROC* [31]. To ensure reproducibility, VIP scores and AUROC values, including the receiver operating characteristic (ROC) curves, were collected through a 5-fold cross-validation, repeated 10 times. Briefly, the 5-fold cross-validation divides the data into five balanced groups using stratified sampling. Four of these groups are used for training the model, while the fifth is used for validation and performance estimation. The procedure is repeated five times, so that each group may act as a test set. Variables with an average VIP score equal to or above 1.0 were seen as significantly altered. Although the analytical approach does not ensure significant changes in the independent metabolites, the direction of change according to difference in group averages was indicated with arrows (↑↓), e.g., ↑ SPMS‒controls refers to an averaged increased level in SPMS patients compared with controls. However, to increase the understanding of the altered metabolites (VIP ≥ 1.0) dependently, all altered metabolites were subjected to a Welch’s *t*-test, which accounts for unequal variance and sample size. The *p*-value adjustment for multiple comparisons was done using false discovery rate (FDR). Only when an FDR value < 0.05 was achieved was the change in mean considered statistically significant.

Altered metabolites with an available KEGG identifier from each model were separately subjected to a pathway analysis using MetaboAnalyst [32] and the *Homo sapiens* pathway library. The significant levels were based on the hypergeometric test and the relative betweenness centrality was used to compute the pathway impact. A *p*-value < 0.05 was considered statistically significant.

Spearman’s ranked correlation analyses were performed between altered metabolite levels and radiological data as well as the EDSS and disease duration reported in months in RRMS and SPMS patients. A correlation with a *p*-value < 0.05 was seen as statistically significant. Hierarchical clustering was performed on the correlation patterns using the R function *hclust* and the Euclidean distance was used as a similarity measure.

## 3. Results

### 3.1. Participant Demographics

This single-center study was conducted on 46 MS subjects (30 RRMS and 16 SPMS patients) and 10 controls with other, non-inflammatory neurological diseases. Seventeen of the RRMS patients and two of the SPMS patients had active gadolinium-enhancing lesions on T1 weighted images, whereas 15 of the RRMS and one of the SPMS patients had ongoing treatment with disease-modifying drugs. The post hoc analysis, comparing treatment status in MS patients, revealed no significant difference between patients with or without ongoing treatments as the model did not achieve predictivity (*Q*^2^ < 0). Follow-up data were available for 40 patients, revealing that four RRMS patients had been diagnosed with SPMS two to three years after sample donation, of whom one had passed away from MS, Table 1.

### 3.2. The CSF Metabolome Could Distinguish SPMS Patients from RRMS and Controls

In total, 117 metabolites with 75% coverage were successfully identified using an *in-house* library; one was removed after investigating associations to the albumin ratio. To account for the potential effects of age on metabolite expression, linear regression models were used to estimate the contribution of age using RRMS patients and controls. In total, 17 (15%) of the identified metabolites were age-dependent and therefore corrected for age in all subjects.

**Figure 1:**
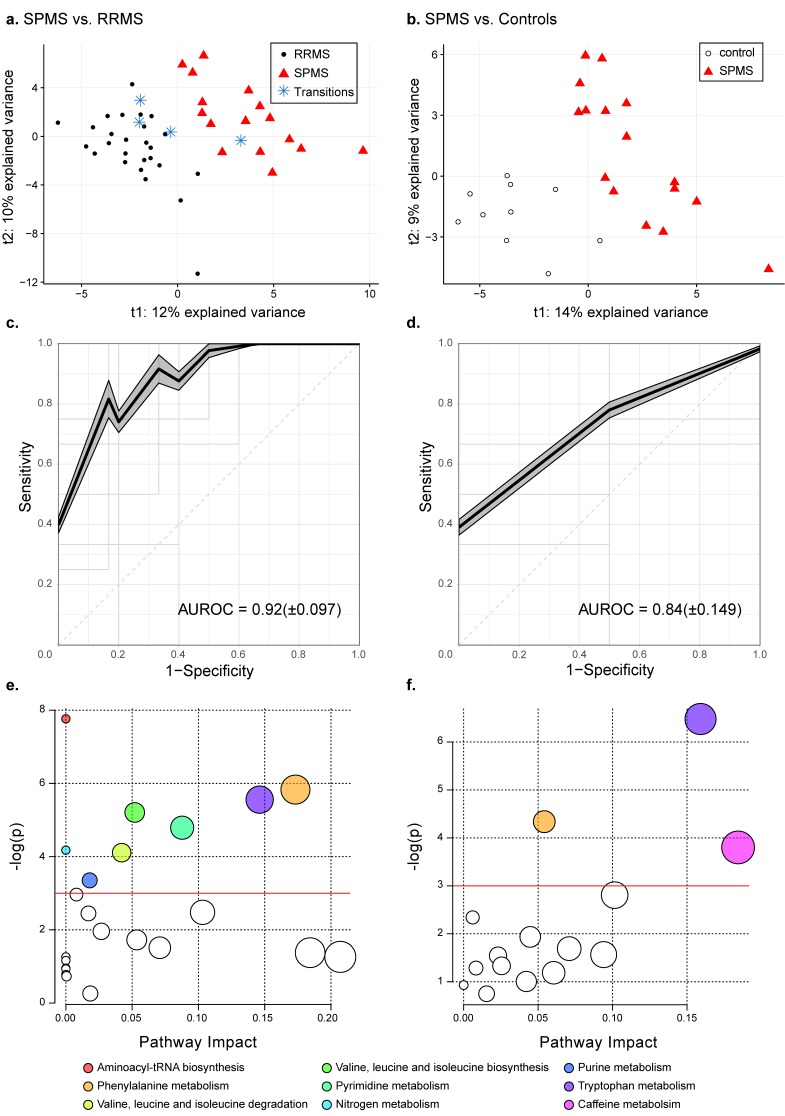
Metabolic differences in SPMS compared with RRMS patients and controls. PLS-DA models comparing (**a**) SPMS vs. RRMS with quality metrics of *R*^2^ = 0.81: *p* < 0.05, *Q*^2^ = 0.47: *p* < 0.05 and (**b**) SPMS vs. controls with quality metrics of *R*^2^ = 0.85: *p* < 0.25, *Q*^2^ = 0.34: *p* < 0.05. The projected transitioning RRMS patients are represented by blue stars. Average ROC curves with corresponding average AUROC and standard deviation for the PLS-DA models comparing (**c**) SPMS with RRMS and (**d**) SPMS with controls. The shadowed areas indicate the standard error of the mean of the sensitivity and 1-specificity. (**e**) Pathway analyses on altered metabolites in SPMS compared with RRMS patients, and (**f**) SPMS patients compared with controls. The size of the node indicates the pathway impact (similar to the x-axis) computed by the relative betweenness centrality and the color corresponds to the pathway. Pathways that were found non-significant in both comparisons have been colored white. The red lines indicate the significance level of *p* = 0.05.

To extract altered metabolites distinguishing the groups, PLS-DA models were trained, comparing two groups each (SPMS vs. RRMS, SPMS vs. controls and RRMS vs. controls). The model comparing the MS phenotypes (SPMS vs. RRMS) (Figure 1a) achieved quality metrics of *R*^2^ = 0.81: *p* < 0.05, *Q*^2^ = 0.47: *p* < 0.05 and an average AUROC of 0.92 (±0.097) (Figure 1c), where the four transitioning patients were kept out of the training and instead projected into the model space. The second model (SPMS vs. controls), Figure 1b, achieved quality metrics of *R*^2^ = 0.85: *p* < 0.25, *Q*^2^ = 0.34: *p* < 0.05 and an average AUROC of 0.84 (±0.149), Figure 1d, whereas comparing RRMS patients with controls showed no significant difference between the groups (*Q*^2^ < 0).

### 3.3. Phenylalanine and Tryptophan Metabolisms Were Altered in SPMS Compared with RRMS Patients

Comparing SPMS with RRMS patients, 37 metabolites achieved averaged VIP scores ≥ 1.0 and were seen as altered, Table 2. The univariate analyses showed that 28 were significantly altered in independence, of which 21 remained significant after correcting for multiple comparisons. The pathway analysis revealed eight biochemical pathways that were affected in SPMS compared with RRMS patients: aminoacyl-tRNA biosynthesis; phenylalanine metabolism; tryptophan metabolism; valine, leucine and isoleucine biosynthesis; pyrimidine metabolism; nitrogen metabolism; valine, leucine and isoleucine degradation and purine metabolism (Figure 1e, Table 3). Complete results from the pathway analysis are reported in Table S2.

### 3.4. Tryptophan Metabolism Were Altered in SPMS Compared with Controls

Comparing SPMS with controls, 32 metabolites were found to be altered between the groups, Table 4. Fourteen of these were independently altered, of which eight remained significant after correcting for multiple comparisons. In the pathway analysis we found three biochemical pathways that were affected in SPMS compared with controls: tryptophan metabolism; phenylalanine metabolism and caffeine metabolism (Figure 1f, Table 5). Complete results from the pathway analysis are reported in Table S3.

Comparing metabolites that were altered in SPMS patients compared with RRMS and controls, 19 metabolites were in common: 1-methyladenosine, 3-methoxytyramine, 4-acetamidobutanoate, 5-hydroxyindoleacetate (5-HIAA), 5-hydroxytryptophan (5-HTP), caffeine, deoxyuridine, guanosine, ketoleucine, kynurenate (KYNA), *N*-acetylleucine, *N*-acetylphenylalanine, *N*-acetylserotonin, *N*6-(delta2-isopentenyl)-adenine, *O*-succinyl-homoserine, phenylacetate, pipecolate, trigonelline and uridine. These biochemical changes represents alterations unique to the SPMS phenotype in comparison with both RRMS and controls.

**Figure 2:**
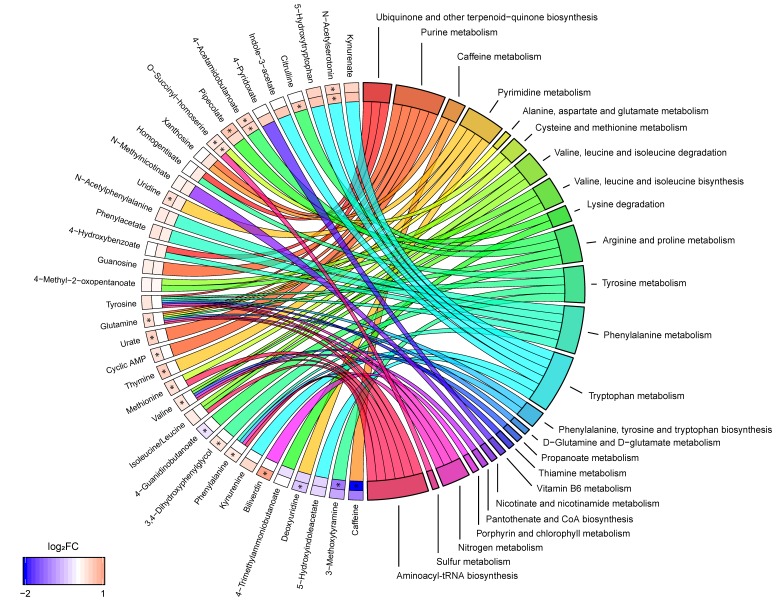
Metabolite to biochemical pathway linkages. The altered metabolites have been linked with pathways as color-coded ribbons. Blue-to-red coding next to the altered metabolites indicates the magnitude and direction of the log^2^ fold change (FC), where the inner layer represents FC in comparison with controls (SP-C) and the outer in comparison with RRMS (SP-RR). Significant FC with an FDR < 0.05 have been marked with a ‘*’.

Combining the results from both pathway analyses revealed that seven biochemical pathways were linked to four or more metabolites: purine metabolism (cyclic AMP, glutamine, guanosine, urate, xanthosine), pyrimidine metabolism (deoxyuridine, glutamine, thymine, uridine), arginine and proline metabolism (4-acetamidobutanoate, 4-guanidinobutanoate, citrulline, glutamine), tyrosine metabolism (3,4-dihydroxyphenylglycol, 3-methoxytyramine, homogentisate, tyrosine), phenylalanine metabolism (4-hydroxybenzoate, *N*-acetylphenylalanine, phenylacetate, phenylalanine, tyrosine), tryptophan metabolism (5-HIAA, 5-HTP, indole-3-acetate, KYNA, kynurenine (KYN), *N*-acetylserotonin, tryptophan) and aminoacyl-tRNA biosynthesis (glutamine, isoleucine/leucine, methionine, phenylalanine, tyrosine, valine) (Figure 2). The metabolites phenylalanine, tyrosine, and glutamine were recurrent in many of these pathways, e.g., these three metabolites comprised half of the metabolites that were linked to aminoacyl-tRNA biosynthesis. Notably, tryptophan metabolism was the pathway linked to the most unique metabolites.

### 3.5. Metabolites Linked to Pyrimidine and Tryptophan Metabolisms Were Associated with Clinical Measurements in MS Patients

**Figure 3:**
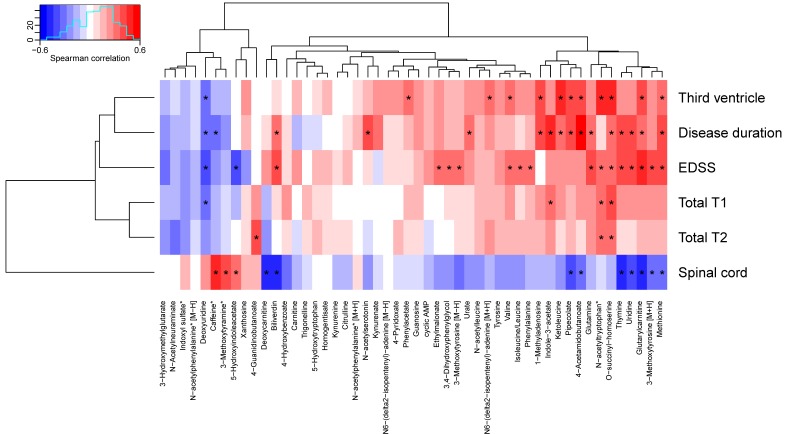
Associations between altered metabolites and clinical data. Metabolite names marked with an asterisk have been corrected for age. Correlations marked with an asterisk are statistically significant (*p* < 0.05). EDSS: Expanded Disability Status Score.

To assess associations between the 50 altered metabolites and clinical measures, the metabolites were associated to radiological data, EDSS, and disease duration, and grouped using hierarchical clustering. Seventeen metabolites depicted significant correlations to EDSS, 16 to disease duration, 12 to the size of the third ventricle, 12 to the size of the spinal cord, four to total T1, and three to total T2. The cluster containing glutamine, *N*-acetyltryptophan, *O*-succinyl-homoserine, thymine, uridine, glutarylcarnitine, 3-methoxytyrosine, methionine, 4-acetamidobutanoate, pipecolate, ketoleucine, indole-3-acetate and 1-methyladenosine depicted multiple significant positive associations to clinical measures as well as a negative association to the size of the spinal cord (see Figure 3, Table 6, and Table S4). *O*-Succinyl-homoserine depicted significant associations with all measures, except the size of the spinal cord, whereas methionine, glutarylcarnitine, deoxyuridine, and *N*-acetyltryptophan demonstrated associations with four clinical measurements, where all three were associated with EDSS and the size of the third ventricle.

The strongest positive associations with disease duration was found for 4-acetamidobutanoate and indole-3-acetate; EDSS: glutarylcarnitine and methionine; the size of the third ventricle: *O*-succinyl-homoserine and ketoleucine; the size of spinal cord: caffeine and 3-methoxytyramine. The strongest negative associations with disease duration were found for deoxyuridine and caffeine; EDSS: deoxyuridine and 5-HIAA; the size of the third ventricle: deoxyuridine; the size of spinal cord: thymine and biliverdin.

In summary, many metabolites including glutamine, thymine, uridine, and deoxyuridine from the pyrimidine metabolism, and indole-3-acetate and 5-HIAA from the tryptophan metabolism displayed associations to multiple clinical measures, but no association with ageing.

## 4. Discussion

There is a current need to understand the molecular basis of the SPMS phenotype. Using HRMS, we have identified and semi-quantified 117 metabolites in CSF that are typically targeted in isolation. By extracting the biochemical differences between SPMS, RRMS, and controls using PLS-DA and connecting these differences to biochemical pathways, we found that multiple pathways, including tryptophan-, phenylalanine-, and pyrimidine metabolism, were altered in SPMS patients. The metabolites phenylalanine, tyrosine, and glutamine were shared between many of these pathways. Thymine, methionine, uridine, deoxyuridine, and glutamine from the pyrimidine metabolism pathway are associated with disability, disease activity, and brain atrophy. These metabolites show no association with ageing, making them of particular interest for understanding the disease mechanisms and as markers of disease progression.

**Figure 4:**
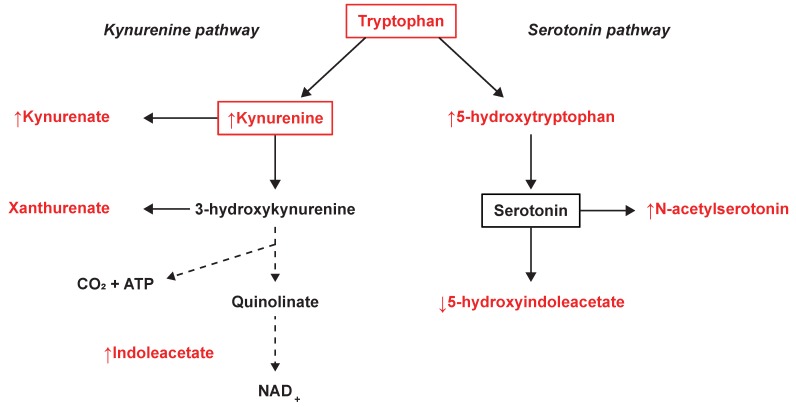
Tryptophan metabolism and the observed changes in the kynurenine and serotonin pathways. The metabolites marked in red were identified and measured, where ↑ illustrates an averaged increase and ↓ an averaged decrease in SPMS patients.

The tryptophan- and phenylalanine metabolism were found to be commonly altered between SPMS and both RRMS patients and controls. Tryptophan metabolism demonstrated strong relevance as it achieved the highest impact in the comparison between SPMS and controls and the second highest in the comparison with RRMS patients. Tryptophan metabolism, and especially metabolites in the tryptophan-degrading kynurenine pathway, has previously been demonstrated to be altered in different stages of MS [11,33]. These metabolites have been recognized as a medium of communication between the immune system and the CNS and may play a central role in the course of the disease [34]. From the kynurenine pathway, KYNA (↑ SP-RR) is directly generated through deamination of KYN (↑ SP-RR, ↑ SP-C) and has been identified as a neuroprotective agent involved in the neurotoxic processes and degenerative mechanisms of MS. Increased levels of KYN have previously been associated with a higher relapse rate in RRMS patients [35]. Elevated levels of KYNA have been found in the plasma of MS patients [36]. Lower KYNA CSF levels have been found in RRMS patients in remission [37], whereas increased levels have been found in acute relapse MS [38]. Herein, we found elevated KYNA levels in SPMS compared with RRMS patients, indicating that the levels change throughout the course of MS and vary between MS phenotypes. Xanthurenate (validated on level 1) from the kynurenine pathway and tryptophan (validated on level 2) were found and identified but not selected as important by the models, suggesting similar levels in SPMS patients compared with RRMS and controls. A summary of the tryptophan-related findings can be found in Figure 4.

From the closely related serotonin pathway, we found significantly increased levels of 5-HTP (↑ SP-RR, ↑ SP-C) and decreased levels of 5-HIAA, (↓ SP-RR, ↓ SP-C) (Figure 4). Also, significantly increased levels of the serotonin derivative *N*-acetylserotonin were found in SPMS compared with RRMS patients and controls, which also demonstrated association with disease duration. Similarly, 5-HIAA demonstrated associations with EDSS and the size of the spinal cord, where the negative correlation with EDSS is in line with previous findings in the CSF of MS patients [39]. The relationship between MS and the serotonergic system (SS), of which serotonin’s main metabolite 5-HIAA is a part, is currently not well understood. Previous studies have shown alterations in the SS in MS patients and underlined the necessity of a deeper characterization of the role of SS in MS pathology [40,41]. Fluoxetine, a candidate drug for repurposing that increases serotonin levels in the CNS, is now clinically tested as a neuroprotective treatment for SPMS patients [42,43]. The neurotransmitter serotonin is known to regulate macrophages, T cells, and dendritic cells and is partially modulated by the gut microbiome [44]. A recently suggested hypothesis propose a connection between an unbalanced microbiota and MS pathology, through SS modulation [41]. This is supported by another recent study emphasizing the potential role of tryptophan metabolism by the gut microbiota in neuroinflammation and MS [35]. Indole-3-acetate (↑ SP-C), which was found to be associated with disease duration and the number of MRI lesions (total T1), has also recently been demonstrated to be a gut microbiota-dependent metabolite. Studies have suggested that indole-3-acetate directly modulates inflammatory responses in hepatocytes and macrophages by attenuation of the release of pro-inflammatory cytokines and induction of the liver to synthesize free fatty acids [45]. These are novel findings and provide a potential link in the host‒microbiota crosstalk and interorgan communication in the tryptophan metabolism that may be of importance for the disease [46].

Pyrimidine-, nitrogen-, and purine metabolism, and valine, leucine, and isoleucine biosynthesis and degradation were uniquely altered between SPMS and RRMS patients. The highest impact was found for pyrimidine metabolism and the four metabolites linked to pyrimidine metabolism were all significantly altered in SPMS compared with RRMS patients, where glutamine (↑ SP-RR), thymine (↑ SP-RR), and uridine (↑ SP-RR, ↑ SP-C) were increased and deoxyuridine was decreased (↓ SP-RR, ↓ SP-C). Furthermore, all demonstrated associations with disease duration and EDSS, where thymine and uridine were also associated with the size of the spinal cord and deoxyuridine with the size of the third ventricle and total T1. The expression of these metabolites showed no association with ageing. Glutamine has previously been reported to be increased in the plasma of MS patients compared with healthy controls [9], and decreasing glutamine brain levels have been reported through disease progression in SPMS patients [47]. The other pyrimidine-linked metabolites have, to the best of our knowledge, not been connected to MS previously.

Pyrimidine metabolism regulates the nucleotide homeostasis through de novo synthesis, catabolism, and nucleotide salvaging and recycling. Nucleotide metabolism has previously been noted to be altered in RRMS patients compared with healthy controls [48]. Furthermore, it is known that pyrimidines play an important role in the modulation of the CNS and alterations in pyrimidine metabolism have been shown in Alzheimer’s disease [49,50]. Pyrimidine synthesis inhibitors are used in treatment of RRMS to block de novo pyrimidine synthesis. These inhibitors interrupt the S phase of the cell cycle in proliferating active T and B cells, limiting their reproduction and involvement in inflammatory processes [51,52]. Thymine, uridine, and deoxyuridine are pyrimidines that would be affected by pyrimidine synthesis inhibitors; however, none of the patients herein were being treated with such drugs.

Taken together, these findings support the importance of pyrimidine metabolism in MS and in particular in the SPMS stage of the disease, and suggest them as potential markers of disease progression.

We found significantly increased levels of methionine (↑ SP-RR) and the methylated adenine residue 1-methyladenosine (↑ SP-RR, ↑ SP-C) in SPMS patients. Methionine is an essential amino acid participating in protein synthesis and the transfer of methyl groups to histones and DNA. Methylation of DNA is known to regulate the expression of thousands of genes and a potential role for epigenetic mechanisms in the course of MS has previously been suggested [53,54,55]. In support of this, dysregulation of methionine metabolism has previously been found in plasma from RRMS patients [56] and post mortem MS brain tissue [57]. Here, we found that methionine had significants association with EDSS, disease duration, and the size of spinal cord and third ventricle, but no association with ageing. These findings support a potential role of epigenetic modifications in MS and suggest that the levels of CSF methionine can be a potential marker of disease progression.

The measurement of identified CSF metabolites in SPMS and RRMS patients, as well as controls, enabled us to develop PLS-DA models with good discriminative power between SPMS and RRMS patients and moderate separation between SPMS patients and controls, which is in line with results based on analysis of metabolites in blood [6,11]. By projecting the transitioning patients into the model space, we found that one of four patients depicted an early biochemical pattern resembling that of an SPMS patient. This detection rate can be improved by adding CSF protein and MRI measurements to the model, as demonstrated by us previously [18].

The most important limitations of the study are the number of patients and the inadequate number of controls that were not age-matched. To correct for the potential effects of ageing on metabolite expression, the age effect was estimated using the RRMS patients and controls by assuming linearity [58], thus covering the age span of SPMS patients. Five of the controls/RRMS patients were above the age of 50 and the oldest patient in the study was an RRMS patient. However, the weakness of this approach is that the linear models will be sensitive for the fewer observations (controls/RRMS patients) present in the higher age span. Moreover, including the SPMS patients in the estimation of ageing would risk removing alterations caused by the neurodegeneration. The general effects of ageing are a challenging confounder in understanding the mechanisms of neurodegenerative diseases [59], and thus we cannot completely rule out the risk that individual metabolite alterations may still be affected by age.

The inter-group post hoc comparisons could rule out gender and treatment status as potential confounding factors. However, even though these factors are not confounding, there may be metabolite expressions that are affected by them. Furthermore, the chance that other potentially underlying confounders that were not investigated or adjusted for are affecting the relationships between metabolite levels and radiological or clinical outcome measures cannot be ruled out either. As such, any novel findings herein need to be replicated in another cohort.

While the limited sample size decreased the statistical power and confidence of our findings, a single-center study design has the advantage of avoiding inter-center variabilities and other confounding factors that can be difficult to correct for [27]. Facing this limitation, a pathway analysis was advantageous as it couples statistical testing to molecular functioning and investigates the metabolites in groups rather than independently [60]. In addition, to ensure reproducibility, cross-validation was performed to more accurately estimate AUROC values and VIP scores of the metabolites. Finally, the metabolites were associated with radiological and clinical measures, which would serve as another level of validation.

Finally, the major challenge in the field of non-targeted metabolomics is metabolite identification [61]. When increasing the number of identified metabolites, it is likely that the discriminative power of the PLS-DA models will increase. Post-identification using an *in-house* library has the advantage of providing highly reliable identities but is limited to the compounds within the library. Although the identified metabolites were able to separate SPMS from RRMS patients and controls, there are more metabolites of interest than those investigated herein.

## 5. Conclusions

Multiple biochemical pathways were altered in SPMS patients, whereof the metabolites phenylalanine, tyrosine, and glutamine were shared between multiple pathways. Metabolites from the kynurenine and serotonin pathways in the tryptophan metabolism were found to be altered in SPMS compared with RRMS patients and controls, indicating a connection between these two pathways and MS pathology, as well as a potential connection with microbiota through the serotonergic system.

Metabolites from the pyrimidine metabolism were all significantly altered between SPMS and RRMS patients and associated with disability, disease activity, and brain atrophy, while showing no association with ageing. This suggests that pyrimidine metabolism and its members are of particular interest for understanding disease mechanisms and as markers of disease progression.

These findings are of importance for the characterization of the molecular pathogenesis of SPMS and support the hypothesis that the CSF metabolome may be used to explore changes that occur in the transition between the RRMS and SPMS pathologies.

## Figures and Tables

**Table 1:** Clinical and demographic data including follow-up data on the patients. Four of the RRMS patients had transitioned to SPMS, one of whom was deceased.

**Cohort**	**Controls**	**RRMS**	**SPMS**
*n*	10	30	16
On treatment, *n*	0	15	1
Age *, mean (±SD)	39 (±13.1)	39 (±10.6)	58 (±9.3)
Female/Male	6/4	21/9	10/6
EDSS *, median(range)	n/a	2.0 (0–7.5)	5.5 (3.0–7.5)
Disease duration *, median (range)	n/a	92 (0.5–364)	283 (109–538)
**Follow up**		**RRMS**	**SPMS**
*n*		27	13
ΔEDSS, median (range)		0.0 (−3.5–3.0)	1.5 (0–4.0)
Time interval in months, mean (±SD)		67.6 (±15.4)	54.8 (±18.6)
Transitioned, *n*		4	n/a
Deceased, *n*		1	0

* Signifies a significant difference between SPMS and RRMS patients. EDSS: Expanded Disability Status Score; n/a: not applicable.

**Table 2:** Altered metabolites with an average VIP score greater than or equal to 1.0 from the PLS-DA comparing SPMS with RRMS patients. A positive log^2^ fold change (FC) SP-RR indicates an averaged increase in SPMS compared with RRMS patients and vice versa. Identities confirmed by *m*/*z* and elution time of the internal standards and by MS/MS fragmentation pattern (validation level 2). Identities confirmed by *m*/*z* and elution time of the internal standards (validation level 1). Coefficient of variation (CV) is reported for all altered metabolites in the QC samples.

Metabolite	KEGG	VIP Mean (95% CI)	log^2^ FC SP-RR	*p*-Value	FDR	CV	Validation Level
Thymine	C00178	2.01 (1.95, 2.07)	0.49	5.0 × 10^−5^	1.8 × 10^-3^	7.9%	1
Glutarylcarnitine	-	1.84 (1.79, 1.90)	0.48	2.4 × 10^−4^	4.4 × 10^-3^	9.3%	2
Biliverdin	C00500	1.78 (1.72, 1.83)	0.90	1.6 × 10^−3^	0.011	24.7%	1
Pipecolate	C00408	1.77 (1.70, 1.83)	0.56	1.9 × 10^−3^	0.011	7.5%	1
Uridine	C00299	1.76 (1.70, 1.82)	0.33	9.4 × 10^−4^	0.011	7.4%	2
4-Acetamidobutanoate	C02946	1.72 (1.67, 1.78)	0.40	2.1 × 10^−3^	0.011	9.2%	2
Deoxyuridine	C00526	1.67 (1.62, 1.72)	−0.50	1.4 × 10^−3^	0.011	13.8%	1
Ethylmalonate	-	1.63 (1.56, 1.70)	0.46	9.7 × 10^−3^	0.030	7.1%	2
Valine	C00183	1.61 (1.56, 1.66)	0.25	4.2 × 10^−3^	0.020	4.5%	2
*O*-Succinyl-homoserine	C01118	1.57 (1.52, 1.62)	0.24	6.4 × 10^−3^	0.021	14.3%	1
Methionine	C00073	1.56 (1.51, 1.62)	0.32	6.0 × 10^−3^	0.021	3.4%	2
Glutamine	C00064	1.51 (1.46, 1.56)	0.31	6.3 × 10^−3^	0.021	22.3%	2
3-Methoxytyrosine [M + H]	-	1.38 (1.31, 1.45)	0.80	0.075	0.088	6.8%	2
Phenylacetate	C07086	1.38 (1.32, 1.43)	0.26	0.031	0.052	8.7%	2
*N*-Acetylleucine	C02710	1.37 (1.33, 1.42)	0.24	0.016	0.038	5.8%	1
Phenylalanine	C00079	1.35 (1.30, 1.39)	0.23	0.021	0.043	4.6%	2
1-Methyladenosine	C02494	1.34 (1.28, 1.39)	0.26	0.017	0.040	11.5%	2
Urate	C00366	1.25 (1.20, 1.31)	0.44	0.013	0.037	8.6%	2
Caffeine *	C07481	1.25 (1.18, 1.32)	−1.12	0.078	0.088	24.4%	2
Ketoleucine	C00233	1.25 (1.19, 1.31)	0.09	0.034	0.052	7.6%	2
Tyrosine	C00082	1.25 (1.21, 1.29)	0.25	0.034	0.052	5.2%	2
*N*6-(delta2-isopentenyl)-adenine	C04083	1.23 (1.17, 1.29)	0.29	0.028	0.049	5.4%	1
*N*-Acetylphenylalanine * [M + H]	C03519	1.23 (1.18, 1.27)	0.21	0.044	0.061	6.9%	1
3-Methoxytyramine *	C05587	1.20 (1.14, 1.26)	−0.75	0.038	0.054	26.7%	1
Cyclic AMP	C00575	1.20 (1.10, 1.31)	0.28	0.020	0.043	12.0%	1
*N*-Acetylserotonin	C00978	1.20 (1.15, 1.25)	0.44	0.023	0.045	16.8%	1
3,4-Dihydroxyphenylglycol	C05576	1.18 (1.13, 1.24)	0.28	0.015	0.038	15.9%	1
Guanosine	C00387	1.17 (1.10, 1.25)	0.16	0.035	0.052	7.8%	2
Kynurenine	C00328	1.12 (1.06, 1.18)	0.37	0.048	0.063	7.9%	2
Isoleucine/Leucine	C00407	1.10 (1.05, 1.15)	0.21	0.078	0.088	6.5%	2
Kynurenate	C01717	1.09 (1.02, 1.16)	0.43	0.050	0.064	9.1%	1
5-Hydroxytryptophan	C00643	1.09 (1.02, 1.15)	0.45	0.055	0.068	15.8%	1
3-Methoxytyrosine [M − H]	-	1.08 (1.05, 1.12)	0.65	0.146	0.150	12.0%	1
4-Guanidinobutanoate	C01035	1.06 (1.01, 1.12)	−0.24	0.028	0.049	7.9%	1
5-Hydroxyindoleacetate	C05635	1.06 (0.99, 1.12)	−0.35	0.083	0.091	11.7%	1
Trigonelline	C01004	1.05 (0.97, 1.12)	0.09	0.157	0.157	11.3%	1
3-Hydroxymethylglutarate	C03761	1.01 (0.94, 1.08)	−0.25	0.107	0.113	13.3%	1

* Metabolite with a demonstrated age dependence that has been corrected. CI: confidence interval.

**Table 3:** Results from the pathway analysis based on the altered metabolites in SPMS compared with RRMS patients.

Pathway	Coverage	*p*-Value	FDR	Impact
Aminoacyl-tRNA biosynthesis	6/56	4.2 × 10^−^^4^	0.034	0
Phenylalanine metabolism	4/45	2.9 × 10^−^^3^	0.103	0.173
Tryptophan metabolism	5/79	3.9 × 10^−^^3^	0.103	0.146
Valine, leucine & isoleucine biosynthesis	3/27	5.5 × 10^−^^3^	0.110	0.052
Pyrimidine metabolism	4/60	8.3 × 10^−^^3^	0.133	0.088
Nitrogen metabolism	3/39	0.015	0.188	0
Valine, leucine & isoleucine degradation	3/40	0.016	0.188	0.042
Purine metabolism	4/92	0.035	0.350	0.018

**Table 4:** Altered metabolites with an average VIP score greater than or equal to 1.0 from the PLS-DA comparing SPMS patients with controls. A positive log^2^ fold change (FC) SP-C indicates an averaged increase in SPMS patients compared with controls and vice versa. Identities confirmed by *m*/*z* and elution time of the internal standards and by MS/MS fragmentation pattern (validation level 2). Identities confirmed by *m*/*z* and elution time of the internal standards (validation level 1). Coefficient of variation (CV) is reported for all altered metabolites in the QC samples.

Metabolite	KEGG	VIP Mean (95% CI)	log^2^ FC SP-C	*p*-Value	FDR	CV	Validation Level
Caffeine *	C07481	1.84 (1.80, 1.88)	−1.97	4.3 × 10^−3^	0.033	24.4%	2
Citrulline	C00327	1.83 (1.76, 1.90)	0.55	5.4 × 10^−3^	0.033	14.2%	1
1-Methyladenosine	C02494	1.80 (1.77, 1.84)	0.40	1.9 × 10^−3^	0.033	11.5%	2
3-Methoxytyramine *	C05587	1.79 (1.72, 1.85)	−1.16	0.012	0.049	26.7%	1
4-Acetamidobutanoate	C02946	1.69 (1.64, 1.74)	0.43	6.1×10^−3^	0.033	9.2%	2
*N*-Acetylserotonin	C00978	1.65 (1.57, 1.73)	0.59	6.2 × 10^−3^	0.033	16.8%	1
*O*-Succinyl-homoserine	C01118	1.64 (1.60, 1.69)	0.28	4.7 × 10^−3^	0.033	14.3%	1
*N*6- (delta2-isopentenyl)-adenine [M + H]	C04083	1.64 (1.59, 1.69)	0.36	9.8 × 10^−3^	0.045	5.4%	1
Trigonelline	C01004	1.59 (1.51, 1.66)	0.20	0.021	0.067	11.3%	1
5-Hydroxytryptophan	C00643	1.47 (1.42, 1.52)	0.57	0.016	0.057	15.8%	1
Kynurenate	C01717	1.37 (1.32, 1.43)	0.60	0.039	0.113	9.1%	1
*N*-Acetylneuraminate	C00270	1.37 (1.29, 1.45)	−0.27	0.062	0.117	7.0%	2
*N*6- (delta2-isopentenyl)-adenine [M − H]	C04083	1.32 (1.26, 1.38)	0.29	0.075	0.126	8.3%	1
*N*-Acetylphenylalanine * [M + H]	C03519	1.32 (1.26, 1.37)	0.23	0.054	0.116	6.9%	1
Deoxyuridine	C00526	1.31 (1.24, 1.37)	−0.37	0.050	0.114	13.8%	1
Homogentisate	C00544	1.28 (1.21, 1.36)	0.21	0.050	0.114	18.6%	1
5-Hydroxyindoleacetate	C05635	1.26 (1.20, 1.32)	−0.38	0.101	0.135	11.7%	1
Pipecolate	C00408	1.24 (1.16, 1.31)	0.37	0.042	0.113	7.5%	1
*N*-Acetylleucine	C02710	1.19 (1.13, 1.26)	0.15	0.145	0.178	5.8%	1
Indole-3-acetate	C00954	1.19 (1.13, 1.24)	0.54	0.066	0.117	12.2%	2
Uridine	C00299	1.17 (1.10, 1.24)	0.19	0.087	0.132	7.4%	2
Indoxyl sulfate *	C08481	1.17 (1.09, 1.25)	−0.35	0.244	0.252	24.0%	2
*N*-Acetyltryptophan *	C03137	1.12 (1.06, 1.18)	0.35	0.058	0.116	4.3%	1
Deoxycarnitine	C01181	1.10 (0.99, 1.20)	−0.27	0.214	0.228	6.8%	1
Xanthosine	C01762	1.09 (1.02, 1.16)	0.26	0.096	0.134	8.8%	1
Phenylacetate	C07086	1.08 (1.00, 1.16)	0.18	0.202	0.222	8.7%	2
Ketoleucine	C00233	1.07 (1.02, 1.11)	0.07	0.083	0.132	7.6%	2
Carnitine	C00318	1.07 (0.97, 1.16)	−0.18	0.166	0.189	5.8%	2
Guanosine	C00387	1.06 (0.99, 1.13)	0.13	0.162	0.189	7.8%	2
*N*-Acetylphenylalanine * [M − H]	C03519	1.03 (0.96, 1.11)	0.13	0.260	0.260	9.3%	1
4-Pyridoxate	C00847	1.02 (0.97, 1.06)	0.48	0.094	0.134	12.2%	1
4-Hydroxybenzoate	C00156	1.02 (0.94, 1.09)	0.17	0.113	0.145	11.5%	1

* Metabolite with a demonstrated age dependence that has been corrected. CI: confidence interval.

**Table 5:** Results from the pathway analysis based on the altered metabolites in SPMS patients compared with controls.

Pathway	Coverage	*p-*Value	FDR	Impact
Tryptophan metabolism	5/79	1.5 × 10^−3^	0.123	0.159
Phenylalanine metabolism	3/45	0.013	0.522	0.054
Caffeine metabolism	2/21	0.022	0.595	0.184

**Table 6:** Association analysis between altered metabolites and clinical data: radiological data, the expanded disability score scale (EDSS) and disease duration. Only metabolites that depicted a significant association with at least one measure have been included.

Metabolite	Spinal Cord	Third Ventricle	EDSS	Disease Duration	Total T1	Total T2
Caffeine *	0.45 ^#^	−0.12	−0.21	−0.33 ^#^	−0.19	−0.14
1-Methyladenosine	−0.14	0.37 ^#^	0.04	0.44 ^#^	0.24	0.18
3-Methoxytyramine *	0.39 ^#^	−0.16	−0.18	−0.23	−0.07	−0.10
4-Acetamidobutanoate	−0.33 ^#^	0.39 ^#^	0.23	0.60 ^#^	0.23	0.10
*N*-Acetylserotonin	−0.27	0.17	0.08	0.39 ^#^	0.04	−0.04
*O*-Succinyl-homoserine	−0.17	0.47 ^#^	0.30 ^#^	0.31 ^#^	0.37 ^#^	0.33 ^#^
*N*6-(delta2-isopentenyl)-adenine [M + H]	−0.16	0.32 ^#^	0.19	0.26	0.16	0.24
Deoxyuridine	0.28	−0.35 ^#^	−0.41 ^#^	−0.34 ^#^	−0.32 ^#^	−0.26
5-Hydroxyindoleacetate	0.35 ^#^	0.03	−0.38 ^#^	0.0	−0.12	−0.01
Pipecolate	−0.41 ^#^	0.38 ^#^	0.21	0.42 ^#^	0.19	0.16
Indole-3-acetate	-0.12	0.26	0.24	0.52 ^#^	0.31 ^#^	0.27
Uridine	−0.42	0.23	0.43 ^#^	0.42 ^#^	0.27	0.11
*N*-Acetyltryptophan *	−0.09	0.45 ^#^	0.34 ^#^	0.12	0.30 ^#^	0.31 ^#^
Deoxycarnitine	−0.45 ^#^	0.03	0.27	0.09	−0.12	−0.20
Phenylacetate	−0.08	0.33 ^#^	0.07	0.16	0.16	0.11
Ketoleucine	−0.25	0.48 ^#^	0.24	0.34 ^#^	0.27	0.11
Thymine	−0.50 ^#^	0.28	0.42 ^#^	0.39 ^#^	0.26	0.08
Glutarylcarnitine	−0.44 ^#^	0.39 ^#^	0.52 ^#^	0.29 ^#^	0.26	0.18
Biliverdin	−0.46 ^#^	0.08	0.41 ^#^	0.32 ^#^	0.12	−0.03
Ethylmalonate	−0.27	0.26	0.31 ^#^	0.25	0.02	0.02
Valine	−0.25	0.30^#^	0.31 ^#^	0.15	0.21	0.13
Methionine	−0.35 ^#^	0.30^#^	0.44 ^#^	0.39 ^#^	0.21	0.16
Glutamine	−0.14	0.27	0.43 ^#^	0.31 ^#^	0.27	0.28
3-Methoxytyrosine [M + H]	−0.33 ^#^	0.27	0.38 ^#^	0.28	0.23	0.16
Phenylalanine	−0.17	0.23	0.35 ^#^	0.14	0.16	0.16
Urate	−0.22	0.26	0.23	0.33 ^#^	0.12	0.10
3,4-Dihydroxyphenylglycol	−0.27	0.16	0.29 ^#^	0.19	0.11	0.01
Isoleucine/Leucine	−0.22	0.24	0.36 ^#^	0.1	0.12	0.15
3-Methoxytyrosine [M − H]	−0.30	0.20	0.33 ^#^	0.14	0.09	0.11
4-Guanidinobutanoate	0.16	0.03	−0.01	0.03	0.23	0.38 ^#^

* Metabolite with a demonstrated age dependence that has been corrected. ^#^ Significant correlation values (*p*-value < 0.05). EDSS: Expanded Disability Status Score.

## Supplementary Materials

The following are available online at http://www.mdpi.com/2073-4409/8/2/84/s1, Table S1: Non-default parameter values used for pre-processing in KNIME. For all parameters not mentioned, the default values were used, Table S2: Complete results from the pathway analysis based on the altered metabolites in SPMS compared with RRMS patients, Table S3: Complete results from the pathway analysis based on the altered metabolites in SPMS patients compared to controls, Table S4: Complete results from the association analysis between altered metabolites and radiological/clinical data.
