# Supplementary material for: Biochemical Differences in Cerebrospinal Fluid between Secondary Progressive and Relapsing–Remitting Multiple Sclerosis
Source: Cells. 2019 Jan 24;8(2):84. doi: 10.3390/cells8020084 (PMC6406712; doi:10.3390/cells8020084)
Supplement: Supplementary file 1 [file cells-08-00084-s001.zip › Supplementary information.pdf]

Biochemical differences in cerebrospinal fluid between secondary progressive and relapsing-remitting multiple sclerosis

Stephanie Herman, Torbjörn Åkerfeldt, Ola Spjuth, Joachim Burman, Kim Kultima

Table S1. Non-default parameter values used for pre-processing in KNIME. For all parameters not mentioned, the default values were used.

Parameter	Value
FeatureFinderMetabo	
chrom_peak_snr	2.0
min_trace_length	1.0
isotope_filtering_model	None
report_convex_hulls	true
FeatureLinkerUnlabeledQT	
nr_partitions	10
ignore_charge	true
max_difference (distance_RT)	10.0
max_difference (distance_MZ)	5.0
unit	ppm

Table S2. Complete results from the pathway analysis based on the altered metabolites in SPMS compared with RRMS patients.

Pathway	Coverage	p-value	FDR	Impact
Aminoacyl-tRNA biosynthesis	6/56	4.2×10^{-4}	0.034	0
Phenylalanine metabolism	4/45	2.9×10^{-3}	0.103	0.173
Tryptophan metabolism	5/79	3.9×10^{-3}	0.103	0.146
Valine, leucine & isoleucine biosynthesis	3/27	5.5×10^{-3}	0.110	0.052
Pyrimidine metabolism	4/60	8.3×10^{-3}	0.133	0.088
Nitrogen metabolism	3/39	0.015	0.188	0
Valine, leucine & isoleucine degradation	3/40	0.016	0.188	0.042
Purine metabolism	4/92	0.035	0.350	0.018
Phenylalanine, tyrosine & tryptophan biosynthesis	2/27	0.052	0.459	0.008
Tyrosine metabolism	3/76	0.084	0.629	0.103
Arginine & proline metabolism	3/77	0.087	0.629	0.017
D-Glutamine & D-glutamate metabolism	1/11	0.141	0.941	0.027
Cysteine & methionine	2/56	0.178	1	0.053
Sulfur metabolism	1/18	0.221	1	0.071
Caffeine metabolism	1/21	0.253	1	0.184
Thiamine metabolism	1/24	0.283	1	0
Alanine, aspartate & glutamate metabolism	1/24	0.283	1	0.207

Pantothenate & CoA biosynthesis	1/27	0.313	1	0
Propanoate metabolism	1/35	0.385	1	0
Ubiquinone & other terpenoid-quinone biosynthesis	1/36	0.394	1	0
Nicotinate & nicotinamide metabolism	1/44	0.458	1	0
Lysine degradation	1/47	0.481	1	5.0×10^{-4}
Porphyrin & chlorophyll metabolism	1/104	0.770	1	0.018

Table S3. Complete results from the pathway analysis based on the altered metabolites in SPMS patients compared with controls.

**Pathway**	**Coverage**	**p-value**	**FDR**	**Impact**
Tryptophan metabolism	5/79	1.5×10^{-3}	0.123	0.159
Phenylalanine metabolism	3/45	0.013	0.522	0.054
Caffeine metabolism	2/21	0.022	0.595	0.184
Ubiquinone and other terpenoid-quinone biosynthesis	2/36	0.060	1	0.101
Lysine degradation	2/47	0.096	1	0.006
Pyrimidine metabolism	2/60	0.144	1	0.045
Sulfur metabolism	1/18	0.184	1	0.071
Tyrosine metabolism	2/76	0.209	1	0.094
Arginine & proline metabolism	2/77	0.213	1	0.023
Valine, leucine & isoleucine biosynthesis	1/27	0.264	1	0.025
Purine metabolism	2/92	0.276	1	0.008
Vitamin B6 metabolism	1/32	0.305	1	0.060
Valine, leucine & isoleucine degradation	1/40	0.366	1	0.042
Nicotinate & nicotinamide metabolism	1/44	0.394	1	0
Cysteine & methionine metabolism	1/56	0.472	1	0.015
